# Early Recognition of Neonatal Septic Arthritis in Premature Infants: Diagnostic Challenges and Clinical Implications

**DOI:** 10.1002/ccr3.72679

**Published:** 2026-05-08

**Authors:** Muhammad Abdullah Awan

**Affiliations:** ^1^ Wah Medical College National University of Medical Sciences Wah Pakistan

Dear Editor,

1

I read with great interest the case report by Arnold et al. describing septic arthritis of the hip in a premature neonate caused by *Staphylococcus aureus* . The report highlights an important but uncommon presentation of neonatal infection and emphasizes the diagnostic difficulties associated with musculoskeletal infections in premature infants.

Septic arthritis in neonates remains a diagnostic challenge because early clinical manifestations are often subtle and nonspecific. Premature infants, in particular, may lack the classic signs of joint infection seen in older children, and symptoms such as irritability, decreased limb movement, or feeding intolerance may initially be attributed to other neonatal conditions. The authors' report therefore underscores the importance of maintaining a high index of suspicion for septic arthritis in neonates presenting with unexplained limb immobility or localized swelling [1].

Another notable aspect of the case is the role of imaging in early diagnosis. Ultrasound and magnetic resonance imaging have become increasingly valuable in detecting joint effusion and early inflammatory changes before radiographic abnormalities appear. Early imaging combined with prompt microbiological evaluation can facilitate timely surgical drainage and targeted antimicrobial therapy, which are critical to preventing complications such as joint destruction, growth disturbance, or long‐term functional impairment [1].

Importantly, this case also raises a broader clinical question regarding early diagnostic triggers in premature neonates with suspected musculoskeletal infection. Given the potential for rapid disease progression and the vulnerability of premature infants to invasive bacterial infections, clinicians may benefit from adopting a lower threshold for early imaging and orthopedic consultation when unexplained limb immobility or swelling is observed. Earlier recognition could potentially reduce diagnostic delay and improve outcomes by enabling prompt intervention.

## Conclusion

This case therefore provides a valuable reminder that neonatal septic arthritis should remain an important differential diagnosis in premature infants presenting with reduced limb movement or unexplained systemic symptoms. Increased awareness among clinicians may facilitate earlier recognition and improved clinical outcomes.

## Author Contributions

**Muhammad Abdullah Awan:** conceptualization, data curation, formal analysis, visualization, writing – original draft, writing – review and editing, supervision, project administration.

## Funding

The author has nothing to report.

## Conflicts of Interest

The author declares no conflicts of interest.

## Data Availability

The data that support the findings of this study are available on request from the corresponding author. The data are not publicly available due to privacy or ethical restrictions.
